# From Waste to Water Purification: Textile-Derived Sorbents for Pharmaceutical Removal

**DOI:** 10.3390/ma17153684

**Published:** 2024-07-25

**Authors:** Magdalena Mazur, Kamyar Shirvanimoghaddam, Moon Paul, Minoo Naebe, Tomasz Klepka, Artur Sokołowski, Bożena Czech

**Affiliations:** 1 Department of Radiochemistry and Environmental Chemistry, Faculty of Chemistry, Maria Curie-Sklodowska University in Lublin, Pl. M. Curie-Sklodowskiej 3, 20-031 Lublin, Poland; magdamazur1993@wp.pl (M.M.); artur.sokolowski@mail.umcs.pl (A.S.); 2 School of Fashion and Textiles, RMIT University, Brunswick, VIC 3056, Australia; kamyar.shirvani.moghaddam@rmit.edu.au; 3 Carbon Nexus, Institute for Frontier Materials, Deakin University, Geelong, VIC 3216, Australia; m.paul@deakin.edu.au (M.P.); minoo.naebe@deakin.edu.au (M.N.); 4 Department of Technology and Polymer Processing, Faculty of Mechanical Engineering, Lublin University of Technology, ul. Nadbystrzycka 36, 20-618 Lublin, Poland; t.klepka@pollub.pl

**Keywords:** adsorption, diclofenac, carbamazepine, cotton-derived biochar, sorption kinetics

## Abstract

The presence of pharmaceuticals or their active metabolites in receiving waters is a sign of the inefficient removal of bioactive substrates from wastewater. Adsorption seems to be the most effective and inexpensive method of their removal. Waste management aimed at sorbents is a promising way to sustain several sustainable development goals. In the presented paper, the removal of the two most widely used drugs in the wastewater was examined. Diclofenac and carbamazepine were removed from water and wastewater using textile waste-derived sorbents. Their removal efficiency was verified by testing several process parameters such as the time of the sorption, the presence of interfering inorganic ions, the presence of dissolved organic matter, the initial pH and ionic strength of the solution, and various water matrices. The adsorption capacity was noted for diclofenac (57.1 mg/g) and carbamazepine (21.25 mg/g). The tested process parameters (pH, presence of inorganic ions, dissolved organic matter, ionic strength, water matrix) confirmed that the presented waste materials possessed a great potential for pharmaceutical removal from water matrices.

## 1. Introduction

Over the last decade, significant attention has been paid to the quality of water and wastewater [1,2,3]. Pharmaceuticals and Personal Care Products (PPCPs) are noted in many environmental matrices including soil [4], surface water [5], and animals [6]. The main introduction route of PPCPs is treated wastewater [7]. Diclofenac (DCF) is a non-steroidal anti-inflammatory over-the-counter drug found even in Antarctic water at the levels of µg/L. Carbamazepine (CBZ) is often used as a psychiatric drug [8]. The increased consumption and usage of drugs result in their increased disposal in wastewater treatment plants. However, the low levels of the removal of DCF and CBZ at wastewater treatment plants (up to 40% for DCF [9] and 25% for CBZ [10], respectively) result from their high bioactivity and low susceptibility to biodegradation [11]. Therefore, in different water matrices, these drugs may be determined: up to 3.6 µg/L of CBZ [12] and up to 13.4 µg/L of DCF [13] were determined in groundwater, affecting all living organisms. This implies that there is a need to develop new effective methods of PPCPs’ removal from water and wastewater [14,15,16].

Among various methods of PPCPs removal from water matrices, photocatalysis [17], osmosis and membrane filtration [18], and ozonation [19] have been found to be very efficient, with advantages and some limitations. Photocatalysis requires additional irradiation, with UV being the most effective [20]. In ozonation, toxic by-products can be formed [21]. Membrane fouling can also lower the removal efficiency [22]. However, adsorption is a method characterized by low cost, high removal rates, and mild operation conditions [23]. Among adsorbents, waste-derived materials have gained attention recently [24,25,26]. The growing amount of produced waste, including organic waste, means that enormous volumes must be managed. A desirable and environmentally friendly approach is to transform waste materials into products that can realize several Sustainable Development Goals [27]. The waste-derived adsorbents may be obtained through several methods, including sol-gel [28], coprecipitation [29], hydrothermal [30], and thermal treating [31]. Pyrolysis is a process of heating feedstock at temperatures above 200 °C in an atmosphere poor in oxygen. High temperatures of pyrolysis result in increased carbon and ash content [32], and the increased surface area of the adsorbents is due to the removal of organic residues from the pores by elevated temperatures [33]. Previous studies have shown that biochar derived from textile waste effectively adsorbs dissolved organic matter [34]. Verifying its usefulness in removing other organics such as PPCPs is interesting. The objective of the presented study was (i) the estimation of the applicability of waste-derived materials in the removal of the selected pharmaceuticals diclofenac and carbamazepine, and (ii) the examination of the factors affecting the removal of tested pharmaceuticals such as the pH or ionic strength of the solution, the presence of inorganic ions or dissolved organic matter, and different water matrices. The results of the present work highlight the potential usage of waste-derived materials in eliminating emerging pollutants while exhibiting sustainable management of this waste.

## 2. Materials and Methods

Textile waste-derived materials were used as adsorbents. Cotton wastes were pyrolyzed at 900 °C, 1100 °C, 1300 °C, and 1500 °C according to the procedure described in [34] and labeled as CT9, CT11, CT13, and CT15. Briefly, cotton wastes prewashed in ethanol and distilled water (to remove impurities) were dried and slow pyrolysis in an argon atmosphere was applied (5 °C/min, dwelling time: 1 h).

For the characterization of the tested adsorbents, surface area analysis was performed using a Quantachrome autosorb using low-temperature nitrogen adsorption to estimate the extent of surface area, as well as pore volume and diameter. Thermogravimetric analysis (TGA) with a TA-Q50 monitored weight loss during air–atmosphere reactions; this was conducted up to 600 °C with a heating rate of 10 °C/min. Raman spectroscopy (Renishaw InVia, Melbourne, Australia) assessed the graphitization degree, while XRD (Philips X’Pert, Sydney, Australia) with Cu Kα radiation identified phases (at 2θ range: 5–60°). The zeta potential after 72 h in water was measured on a Zetametr Zeta Plus Bi-Mass (Brookhaven Instruments Corporation, Holtsville, NY, USA).

For the adsorption studies, diclofenac sodium salt and carbamazepine (Sigma-Aldrich, Warsaw, Poland), representatives of drugs widely noted in environmental matrices, were used. Different DCF and CBZ solutions in distilled water (CBZ was dissolved initially in methanol 1 vol.% then diluted with distilled water) were used: 20 mg/L for kinetics and 0–100 mg/L for isotherm modeling.

The equilibrium sorption capacity was calculated using Equation (1):

(1) $$Q_{e}=\frac{\left(C_{0}-C_{e}\right)*V}{m}$$

where *Q_e_* is an equivalent concentration of adsorbed DCF or CBZ (mg/g); *C*_0_ and *C_e_* are initial and equivalent concentrations of DCF and CBZ (mg/L), *V* is sample volume (L) and *m* is the mass of the adsorbent (g).

The process of sorption was performed in Falcone tubes (50 mL), using 20 ± 0.1 mg of the adsorbent in contact with the tested adsorbate (mixed at 120 rpm) at room temperature, 23 ± 1 °C. Sorption kinetics were estimated using 20 mg/L of DCF or CBZ and 20 ± 0.1 mg of the adsorbent (mixed at 120 rpm), and the samples were analyzed at the following time intervals: 5, 15, 30, 60, 120 min, and 24 h. For the description of sorption kinetics, two mathematic models were applied: pseudo-first-order (PFO), pseudo-second-order (PSO), Elovich (E), and Intraparticle Diffusion model (IPD), where the values of *k*_1_, *k*_2_, *α*, and *k_IPD_* were obtained from the respective linear dependences [35].

In the PSO model (2),

(2) $${log(Q}_{e}-Q_{t})=\log Q_{e}-\frac{k_{1}}{2.303}t$$

where *Q_e_* and *Q_t_* are the amounts of DCF or CBZ adsorbed at equilibrium and after time *t* (mg/g). The value of *k*_1_ was obtained from the linear relationship (3):

(3) $${log(Q}_{e}-Q_{t})=f(t)$$

The pseudo-second-order reaction (PSO) kinetic model describes (4)

(4) $$\frac{t}{Q_{t}}=\frac{1}{k_{2}Q_{e}^{2}}+\frac{1}{Q_{e}t}$$

in which *k*_2_ is the adsorption rate constant of the pseudo-second-order adsorption rate (g/mg min). The *k*_2_ values were obtained from the linear dependency of *t/Q_t_* vs. *t*. In the Elovich model (5),

(5) $$\frac{{dQ}_{e}}{dt}=\alpha e^{-\beta Q_{t}}$$

where *α* is the initial adsorption rate and *β* is the ratio between the surface coverage and the activation energy. In the Intraparticle Diffusion model (IPD) model (6),

(6) $$Q_{e}=k_{IPD}\sqrt{t}+C$$

where *K_IPD_* is the rate constant for intraparticle diffusion (mg/g min^1/2^), t is the time (min), and C is the intercept.

The filtered samples (0.45 μm syringe filters) were analyzed for DCF or CBZ presence using UV-VIS spectroscopy (Specord 200, Analytik, Jena, Germany; scanning speed 600 nm/min; response time 0.1 s; spectral band 2 nm), with detection wavelengths of λ_DCF_ = 274 nm and λ_CBZ_ = 287 nm. The amount of adsorbed drug was calculated using calibration curves (R^2^_DCF_ = 0.9973 and R^2^_CBZ_ = 0.9942) based on the concentration loss in the aqueous phase.

For the isotherms modeling four nonlinear models, the Langmuir (L) (describing monolayer adsorption), Freundlich (F) (adsorption onto heterogeneous surfaces) [34], Temkin (considering the heat of adsorption and the interaction between the adsorbent and adsorbate [36]) and Dubinin–Radushkevich (Gaussian energy distribution onto heterogeneous surfaces) models were used [37]. The tests were performed in Falcone tubes (50 mL), using 20 ± 0.1 mg of the adsorbent in contact with 0–100 mg/L of tested adsorbate mixed at 120 rpm at room temperature, 23 ± 1 °C. It should be noted that the described systems, which involve ion exchange sorption rather than the physical adsorption of a monomolecular adsorbate layer on the adsorbent surface, do not meet the assumptions of the Langmuir adsorption model. However, despite the obvious departure from the assumptions of this model, the presented method allows for obtaining a satisfactory approximation of adsorption isotherms determined experimentally, and is recommended for the practical modeling of the adsorption equilibrium of the considered systems as a much simpler method than others.

In the Langmuir model (7),

(7) $$\frac{1}{c_{s}}=\frac{1}{Q_{L}K_{L}}\frac{1}{c_{e}}+\frac{1}{Q_{L}}$$

where *Q_L_* is the maximum adsorbed amount of DCF or CBZ (mg/g), and *K_L_* is the sorption equilibrium constant (L/mg). The Freundlich model describes multilayer adsorption onto heterogeneous surfaces (8),

(8) $$\log c_{s}=n\log c_{w}+\log K_{F}$$

where *K_F_* is the relative adsorption capacity (mg/g), and *n* is the linearity parameter.

In the Temkin model (9),

(9) $$lnc_{s}=\frac{RT}{b}lnc_{e}+\frac{RT}{b}lnA$$

where *R* is the universal gas constant, *T* is the absolute temperature, *b* is the heat of adsorption, and *A* is the binding constant (L/mg). In the Dubinin–Radushkevich model (10),

(10) $$lnc_{s}=\log Q_{DR}-\frac{R^{2}T^{2}}{{2E}^{2}}\log^{2}(1+\frac{1}{c_{e}})$$

where *Q_DR_* is the adsorption capacity (mg/g), *B_D_* is the mean free energy of sorption, and *E* is the bonding energy (J/mol) for the ion-exchange mechanism, calculated using Equation (11),

(11) $$E=\frac{1}{\sqrt{{2B}_{D}}}$$

The effects of several process parameters were estimated in Falcone tubes (50 mL), using 20 ± 0.1 mg of the adsorbent in contact with 20 mg/L of tested adsorbate mixed at 120 rpm at room temperature, 23 ± 1 °C. The effect of the initial pH of the solution (4–10) was examined using 0.1 M NaOH or HCl (POCh, Gliwice, Poland). Here, 0–0.2 mM NaCl (POCh, Gliwice, Poland) was used to evaluate the effect of the ionic strength of the solution on DCF or CBZ removal. The effect of the presence of inorganic ions was verified using the 10^−3^ M salts NaCl, NaNO_3_, and Na_3_PO_4_ (POCh, Gliwice, Poland). Tannic acid (Sigma-Aldrich, Poland), as the representative of dissolved organic matter, was applied at 0–100 mg/L concentrations. Different water matrices, distilled water, tap water, and surface water (Bystrzyca River, Lublin, Poland), were examined. All data are expressed as the mean ± standard deviation of three replicates. Single-factor analysis of variance (ANOVA) was used to analyze the data. Probability values at *p* < 0.05 were considered statistically significant.

## 3. Results and Discussion

**Figure 1:**
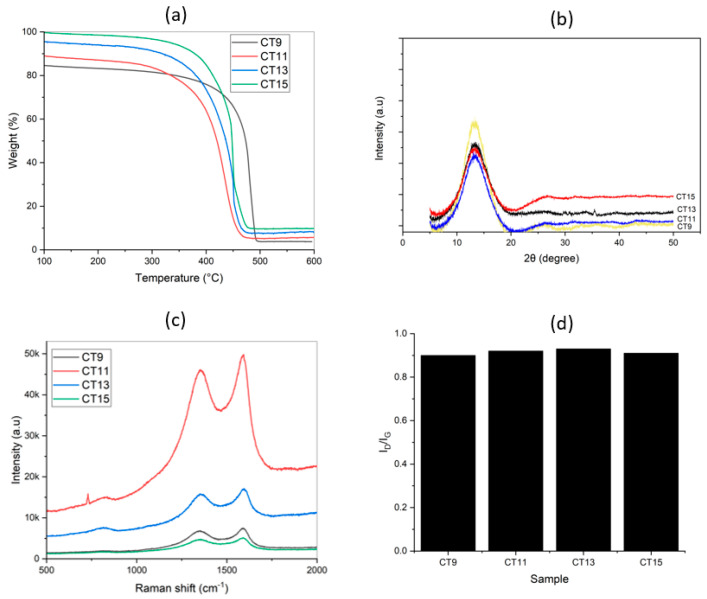
TGA in air (**a**), XRD (**b**), and RAMAN results (**c**,**d**) of CT samples.

**Figure 2:**
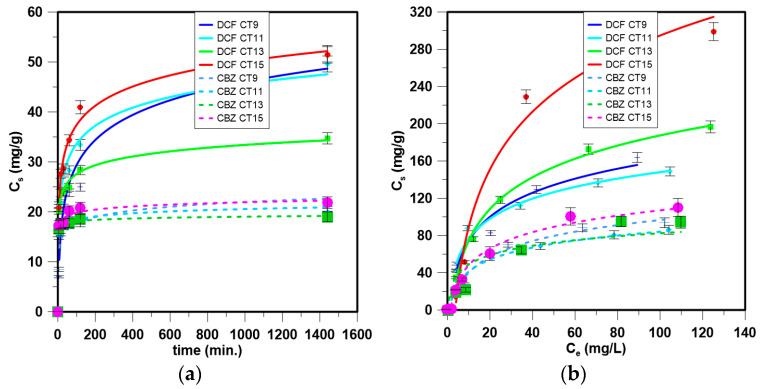
(**a**) Kinetics and (**b**) isotherm modeling of DCF and CBZ adsorption onto tested materials.

The main physicochemical properties of the tested materials are presented in Figure 1. The results of the sorption studies are shown in Table 1, Table 2 and Table 3 and Figure 2.

### 3.1. Physicochemical Characteristics of Tested Materials

The surfaces of the materials were characterized by large surface areas of 533.5, 388.6, 426.1, and 406 m^2^/g, for CT9, CT11, CT13, and CT15, respectively. The mean pore diameter was estimated at 2.5–5 nm [34] and the zeta potential lay in the negative region (−6, −5, −6, and −3 mV, for CT9, CT11, CT13, and CT15, respectively), implying that in water the surface of CT is negatively charged. The samples showed a very good thermal stability after pyrolysis, confirming the great potential for environmental applications (Figure 1a). This indicates the materials can withstand high temperatures without significant degradation, a crucial feature for many environmental applications. In terms of crystallography, the samples represent the common peaks of carbon, as expected; however, in the case of CT15, due to higher pyrolysis temperature, the samples showed higher intensity of the peaks, especially around 2θ = 26° (graphite reference pattern JCPDS no. 00-041-1487) (Figure 1b). This suggests a higher degree of graphitization in CT15 compared to other samples, potentially due to the more extreme thermal treatment.

The Raman results show that the ID/IG changes slightly for different samples, but the amounts of change are negligible (Figure 1c,d), suggesting the efficient graphitization of all samples independent of pyrolysis temperature, which may favor the adsorption of tested compounds. The results imply that even low temperatures of pyrolysis may be suitable for obtaining well-graphitized adsorbents.

### 3.2. Adsorption Kinetics

The results of the adsorption studies are presented in Figure 2 and Table 1. The sorption kinetics of both tested compounds proceeded similarly (Figure 2a). The obtained materials were effective in the adsorption of tested pollutants (Table 1 and Table 2). The highest adsorption amount was noted for DCF, obtaining over CT9—51.67 mg/g and CT15—51.70 mg/g. Diclofenac is a compound that under the tested conditions (pH ≈ 6.4) was ionized, thus ionic substances were adsorbed onto the tested materials preferentially. After 120 min, almost 80% of the maximum adsorption capacity of CT11 and CT15 for DCF was noted. The lowest adsorption of DCF was observed using CT13. The adsorption of CBZ was significantly lower, and up to 21.25 mg/g was adsorbed onto CT9. As in the case of DCF, the first step of adsorption was quick, and after 90 min, almost 90% of the maximum adsorption capacity was estimated. Slower adsorption was maintained using CT9, the material with the highest surface area, implying that the extent of surface area was a key factor affecting CBZ adsorption on cotton-waste-derived adsorbents. An effect of surface area was not noted on DCF adsorption.

The best fitting of the obtained results was noted using the PSO model (R^2^ > 0.994) (Table 1), which implies chemisorption as the rate-limiting step. Chemisorption governed the process in the case of DCF and CBZ; however, higher fitting values were noted for CBZ adsorption (R^2^ > 0.9997) than DCF. PSO is the most widely observed regime of the sorption kinetics of DCF and CBZ onto carbonaceous materials [38].

All tested materials were efficient in removing DCF and CBZ compared to other materials presented in the literature (Table 2), and may be recommended for removing both compounds from water.

**Table 2:** Comparison of the obtained results with the data from the literature.

Sorbent	Compound	Fitting Isotherm	Q_e_ (mg/g)	References
cotton-derived carbon	DCF	Temkin	51.7	This study
activated carbon KOH from red pepper (*Capsicum annuum* L.)	DCF	-	196.1	[39]
rice husk ash-derived biochar	DCF	Freundlich	2.316	[40]
(poly(acrylic acid) (PAA) and poly(sodium methacrylate) (PMAA)), with poly(ethyleneimine) (PEI)	DCF	-	109.94	[41]
Sludge-derived hydrochar	DCF	Langmuir	37.23	[42]
Palygorskite Clays	DCF	Langmuir	253.34	[43]
sycamore ball-activated carbon	DCF	Langmuir	178.89	[38]
cotton-derived carbon	CBZ	Temkin	21.25	This study
pistachio shell composite of L@PSAC	CBZ	Langmuir	>99% from 50 mg/L	[44]
ZnO nanoparticles derived from neem (Azadirachta indica) leaves	CBZ	-	27.55	[45]
nitrogen-doped tantalum carbide	CBZ	Dubinin–Radushkevich	119	[46]

Regarding the rate-limiting step of adsorption, it was noted that the PSO regime better fit the experimental data (R^2^ > 0.9999), stressing that chemisorption is a process that governs the adsorption of CBZ onto tested adsorbents.

### 3.3. Isotherm Modeling

When considering the adsorption mechanism and fitting to the tested models, it can be observed that the process is very complex (Table 3, Figure 2b). The adsorption of DCF by each of the tested materials followed several regimes. Monolayer adsorption (Langmuir model) was observed for the adsorption of DCF onto CT13 and CBZ onto CT15. The Freundlich model fit well to the experimental data obtained for DCF adsorption onto CT9 and CBZ onto CT13. The Temkin model describes the adsorption of DCF onto CT11 and CT15, and CBZ onto CT9 and CT11. The lowest fitting was observed for the D-R adsorption model.

**Table 3:** Isotherm modeling parameters for the adsorption of DCF and CBZ onto the tested materials.

	L				F			T			DR		
	Q_L_	K_L_	R^2^	R_L_	K_F_	*n*	R^2^	Q_T_	B	R^2^	Q_D_	E	R^2^
	[mg/g]	[L/g]	[-]	[-]	[mg/g]	[-]	[-]	[mg/g]	[kJ/mol]	[-]	[mg/g]	[kJ/mol]	[-]
DCF													
CT9	149.37	0.1042	0.9604	0.0604	24.849	0.423	0.9872	0.7354	66.548	0.9599	123.822	374.596	0.8472
CT11	169.92	0.0795	0.9637	0.0689	30.481	0.357	0.8876	1.1792	79.838	0.9645	129.060	338.997	0.9490
CT13	222.52	0.0457	0.9989	0.0896	19.672	0.508	0.9620	0.4565	50.492	0.9933	142.527	320.682	0.8169
CT15	399.61	0.0092	0.9416	0.2138	6.328	0.863	0.9212	0.2717	27.755	0.9837	234.447	224.110	0.9166
CBZ													
CT9	100.69	0.0617	0.9566	0.139	10.789	0.497	0.9621	0.5143	101.06	0.9784	73.664	326.974	0.7613
CT11	100.82	0.0611	0.9792	0.140	12.415	0.432	0.9643	0.6827	122.35	0.9975	76.136	305.481	0.9568
CT13	86.98	0.0611	0.8743	0.158	8.111	0.544	0.9690	0.3889	96.62	0.9652	68.840	319.425	0.6503
CT15	126.36	0.051	0.9985	0.134	12.033	0.501	0.9743	0.4902	87.44	0.9896	83.485	317.451	0.8256

Under monolayer adsorption, as assumed in the Langmuir model, the adsorptions of DCF onto CT13 and CBZ onto CT15 were distributed uniformly over the adsorbent surface. The Freundlich model describes adsorption onto heterogeneous surfaces [47]. Due to the value of the *n* parameter, it can be stated that the most heterogeneous surface was CT11 (*n* = 0.357). In the case of the other materials, the sorption characteristic was rather independent of surface heterogeneity. The Temkin model revealed the best fitting when CT11 was used for the adsorption of both tested compounds, CT9 for CBZ and CT15 for DCF. In this model, the adsorption heat of all molecules decreases linearly with the increase in coverage of the adsorbent surface, and adsorption is characterized by a uniform distribution of binding energies, up to a maximum binding energy [48]. The highest values of binding energies were noted for CBZ. In the Temkin model, b constants were positive, which implies unfavorable thermodynamic adsorption between the adsorbates and activated carbon. From the Temkin isotherm, a reversible adsorption process for CBZ [49] was observed. Chemisorption was confirmed by the low B values in the Temkin model (below 10 kJ/mol) [50]. The obtained data indicate that the processes of both drugs’ adsorption onto tested materials are very complex and cannot be described by one mechanism.

### 3.4. The Effects of Water Parameters on DCF and CBZ Adsorption onto CT

**Figure 3:**
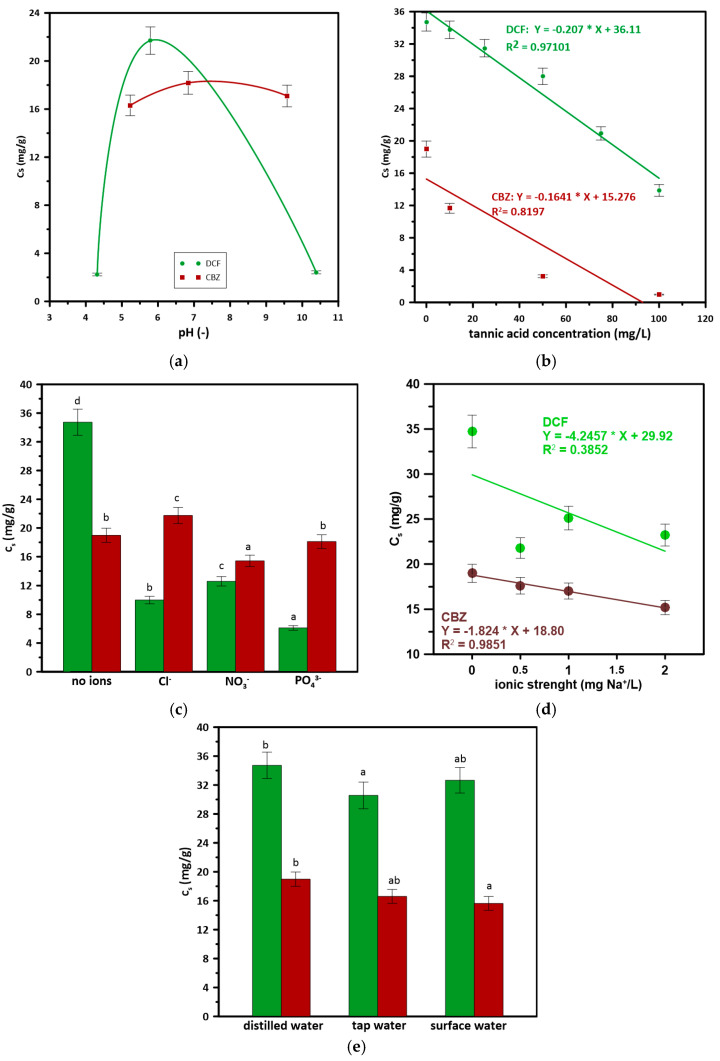
The effects of (**a**) pH, (**b**) DOM, (**c**) inorganic ions, (**d**) ionic strength, and (**e**) water matrix on CBZ and DCF adsorption. Values are presented as mean ± standard deviation (*n* = 3), while lowercase letters (a–d) indicate treatments showing significant differences (*p* < 0.05).

The adsorption of DCF was significantly affected by the pH of the DCF solution (Figure 3a); the pKa of DCF is 4, so at the pH of the sorption surface, DCF will be negatively charged. Additionally, CBZ was present as the neutral compound under the testing conditions [51]. Thus these data indicate that the sorption of negatively charged molecules was favored over that of neutral ones. As CBZ is not dissociated under operating conditions, electrostatic attraction cannot explain its adsorption capacity. Therefore, the other mechanisms may be responsible for CBZ adsorption: hydrogen bonding or π–π interactions [52].

The adsorption was affected by various water constituents, such as inorganic and organic compounds, which may hinder the adsorption simply via competition with the target pollutant for the adsorption site on the sorbent surface. However, in some cases, increased sorption may be observed, especially when water additives mediate between the sorbent surface and pollutants throughout the formation of new active sites [53]. In general, the addition of DOM hindered the adsorption (Figure 3b). In the environment, the content of DOM may reach 10 mg/L, and at that concentration, the adsorption of DCF was hindered by only 3%; however, some linear effect could be noted. This may imply that the surface of CT adsorbs TA more preferentially than hydrophobic CBZ.

Inorganic ions, due to their mobility in the environment, are common components of natural water and wastewater [54]. The most common inorganic ions such as chlorides, nitrates, or phosphates may compete with drugs on an adsorbent surface. Chlorides may hinder the adsorption of DCF, but slightly increase the adsorption of CBZ. PO_4_^3−^ showed no effect on CBZ sorption, whereas it significantly reduced the adsorption of DCF. Nitrates were effective in reducing the amount of adsorbed DCF (Figure 3c). This may be due to the accumulation of a greater number of active sites for a given mass of adsorbents, as was noted in [55]. Interestingly, the presence of Cl^−^ or NO_3_^−^ did not lower CBZ adsorption significantly. An increase in the ionic strength of the drug solution lowered the adsorption of both compounds (Figure 3d); however, a linear (R^2^ = 0.9852) correlation between the amount of adsorbed CBZ and ionic strength was noted. It was noted by Bui and Choi [56] that a higher ionic strength may lower the solubility of neutral pharmaceuticals, but induce the dissolution of ionizable acidic pharmaceuticals. It was observed that the water matrix did not have a significant effect on the adsorption onto tested materials. DCF adsorption was slightly hindered in tap water (Figure 3e), which may imply the presence of Fe in water and water hardness. Simultaneously, the lower adsorption of CBZ in river water may be related to the presence of organics in water.

## 4. Conclusions

The adsorption of the tested drugs onto a waste-derived adsorbent was efficient. The highest adsorption capacity was noted for diclofenac (57.1 mg/g), this being two times higher than that obtained for carbamazepine (21.25 mg/g). The adsorption of DCF and CBZ on CT was limited by the chemisorption of the pharmaceuticals on the surfaces of adsorbents (pseudo-second-order model), but different mechanisms describe the adsorption of DCF and CBZ onto tested materials. The process was enhanced in a slightly acidic medium (the optimal pH of the adsorption process was 5.79 for DCF and 6.84 for CBZ). The presence of soluble organic matter reduced the adsorption rate, which was especially notable in the case of CBZ. Considering inorganic ions, phosphates inhibited adsorption to the greatest extent, while the presence of chlorides or nitrates did not inhibit adsorption significantly. The increase in the ionic strength of the solution inhibited the adsorption process. It is worth stressing that the water matrix did not affect the adsorption significantly. The results strongly imply that cotton-waste-derived adsorbents can be used for the removal of various pharmaceuticals from water and wastewater.

## Figures and Tables

**Table 1:** Kinetic parameters of tested sorbents.

CT		PFO			PSO			Elovich			IPD		
	Q_e_ [mg/g]	k_1_ [min^−1^]	Q [mg/g]	R^2^	k_2_	Q [g/mg min]	R^2^	A	β	R^2^	K_IPD_ [mg/g min^1/2^]	b	R^2^
DCF													
CT9	51.67	0.002	3.607	0.9884	2.547	53.62	0.9944	4.210	0.138	0.9320	1.044	12.993	0.9097
CT11	49.60	23.250	3.267	0.9933	4.275	131.58	0.9986	33.05	0.192	0.9487	0.760	21.724	0.9453
CT13	34.72	0.415	2.467	0.9870	0.047	35.06	0.9997	1371.8	0.395	0.9725	0.354	21.973	0.8909
CT15	51.70	3.274	1.473	0.8298	0.010	12.99	0.9997	47.92	0.181	0.9800	0.737	25.574	0.8150
CBZ													
CT9	21.25	0.003	1.740	0.9251	0.936	21.39	0.9999	258.484	0.539	0.7187	0.212	14.131	0.4389
CT11	20.02	27.292	0.703	0.7066	2.441	131.58	0.9999	>10,000	1.092	0.8262	0.104	16.694	0.5022
CT13	18.98	0.459	0.316	0.9373	0.052	19.01	0.9999	>10,000	2.519	0.8881	0.049	17.324	0.6282
CT15	17.70	3.274	1.473	0.8298	0.010	12.99	0.9997	>10,000	1.094	0.8586	0.116	17.930	0.6479

## Data Availability

The data presented in this study are available upon request from the corresponding author.
